# Understanding Spreading Depression from Headache to Sudden Unexpected Death

**DOI:** 10.3389/fneur.2018.00019

**Published:** 2018-02-01

**Authors:** Olga Cozzolino, Maria Marchese, Francesco Trovato, Enrico Pracucci, Gian Michele Ratto, Maria Gabriella Buzzi, Federico Sicca, Filippo M. Santorelli

**Affiliations:** ^1^NEST, Istituto Nanoscienze CNR and Scuola Normale Superiore, Pisa, Italy; ^2^Molecular Medicine and Clinical Neurophysiology Laboratories, Department of Developmental Neuroscience, IRCCS Fondazione Stella Maris, Pisa, Italy; ^3^Headache Centre and Post-Coma Unit IRCCS, Fondazione Santa Lucia, Rome, Italy

**Keywords:** spreading depression, spreading depolarization, migraine, ischemia, subarachnoid hemorrhage, epilepsy, sudden unexpected death in epilepsy

## Abstract

Spreading depression (SD) is a neurophysiological phenomenon characterized by abrupt changes in intracellular ion gradients and sustained depolarization of neurons. It leads to loss of electrical activity, changes in the synaptic architecture, and an altered vascular response. Although SD is often described as a unique phenomenon with homogeneous characteristics, it may be strongly affected by the particular triggering event and by genetic background. Furthermore, SD may contribute differently to the pathogenesis of widely heterogeneous clinical conditions. Indeed, clinical disorders related to SD vary in their presentation and severity, ranging from benign headache conditions (migraine syndromes) to severely disabling events, such as cerebral ischemia, or even death in people with epilepsy. Although the characteristics and mechanisms of SD have been dissected using a variety of approaches, ranging from cells to human models, this phenomenon remains only partially understood because of its complexity and the difficulty of obtaining direct experimental data. Currently, clinical monitoring of SD is limited to patients who require neurosurgical interventions and the placement of subdural electrode strips. Significantly, SD events recorded in humans display electrophysiological features that are essentially the same as those observed in animal models. Further research using existing and new experimental models of SD may allow a better understanding of its core mechanisms, and of their differences in different clinical conditions, fostering opportunities to identify and develop targeted therapies for SD-related disorders and their worst consequences.

## Introduction

Transient brain dysfunctions characterize several neurological disorders, leading to episodic manifestations, e.g., headache or epileptic seizures. In most transient neurological disorders, the complex pathophysiological processes underlying the occurrence of neuronal and/or glial breakdown and subsequent functional recovery are largely unknown; however, the spreading depression (SD) phenomenon is undoubtedly one of the main ones and seems to play a crucial role in several conditions, primarily ischemia, seizures, migraine, and migraine variants.

The term “spreading depression” indicates slowly propagating changes in neuronal electrical potentials, coinciding with or leading to a silencing of brain electrical activity. The phenomenon self-propagates as a wave in the gray matter by means of contiguity, regardless of functional divisions or arterial territories (1). The first description of SD appeared in 1944 in a study showing a spreading decrease of excitability in experimental epilepsy (2). Numerous studies have further investigated this phenomenon in normal and pathological conditions, with the aim of reaching a better understanding of its involvement in clinical conditions like migraine, ischemic and traumatic brain injury (TBI), transient global amnesia, and epilepsy. However, it remains incompletely understood due to the complexity of synaptic physiology and the difficulty in obtaining direct experimental data. Moreover, it is still debated whether the spreading phenomenon should more properly be considered to refer to the depression or to the depolarization, even though the two propagates in the tissue together (3–5). Some authors use “spreading depolarization” as a generic term indicating the biophysical mechanism underlying the progressive, self-propagating, and ultimately near-complete neuronal depolarization (6), and “spreading depression” to refer to the silencing of brain electrical activity, considered a consequence or epiphenomenon of the spreading depolarization. Others, instead, use the terms “spreading depression” and “spreading depolarization” according to the presence, respectively, of normal or impaired local metabolic conditions when the phenomenon occurs (7, 8). Differences in terminology arise from the evidence that there exist different types of propagating depolarization characterized by heterogeneous molecular signatures. Indeed, pharmacological inhibition of the presumed molecular pathways gives different results according to the initial metabolic condition (8). The purpose of this review is to explain why these phenomena may be regarded as the pathophysiological correlates of different disorders, and from this perspective we consider the first definition less confusing. Therefore, in the present review, the term SD is taken to refer to both spreading phenomena: the propagating depolarization, understood as the initial phenomenon, and the SD proper, understood as its ultimate consequence or epiphenomenon.

## Mechanisms of SD

Neurons use electrochemical energy to drive signaling in the brain. They store this energy in the form of ion gradients across the cell membrane, and the main ions involved in neuronal excitability are sodium, potassium, and chloride. An electrical signal in a neuron requires only a small amount of this energy (9). During an action potential, the flux of very few ions through specific membrane channels causes the membrane potential to rise and fall within the space in a millisecond (10). The activity of the Na/K-ATPase pump is crucial in restoring ion homeostasis. The brain consumes about 20% of the body’s basal energy (11) and Na/K pumps use about half of this 20% share in maintaining ionic gradients.

Glial cells also play a critical role, serving to buffer K^+^ ions in the extracellular space and absorb the glutamate released from excitatory synapses, thereby avoiding excitotoxicity reactions. However, when the concentration of these molecules exceeds a certain threshold, neuronal and glial transporters can no longer cope with the efflux. This results in massive extracellular accumulation of potassium and glutamate and in large cell depolarization accompanied by loss of membrane resistance and large shifts in the intra- and extracellular ion concentrations, preventing the generation of action potentials (12).

**Figure 1:**
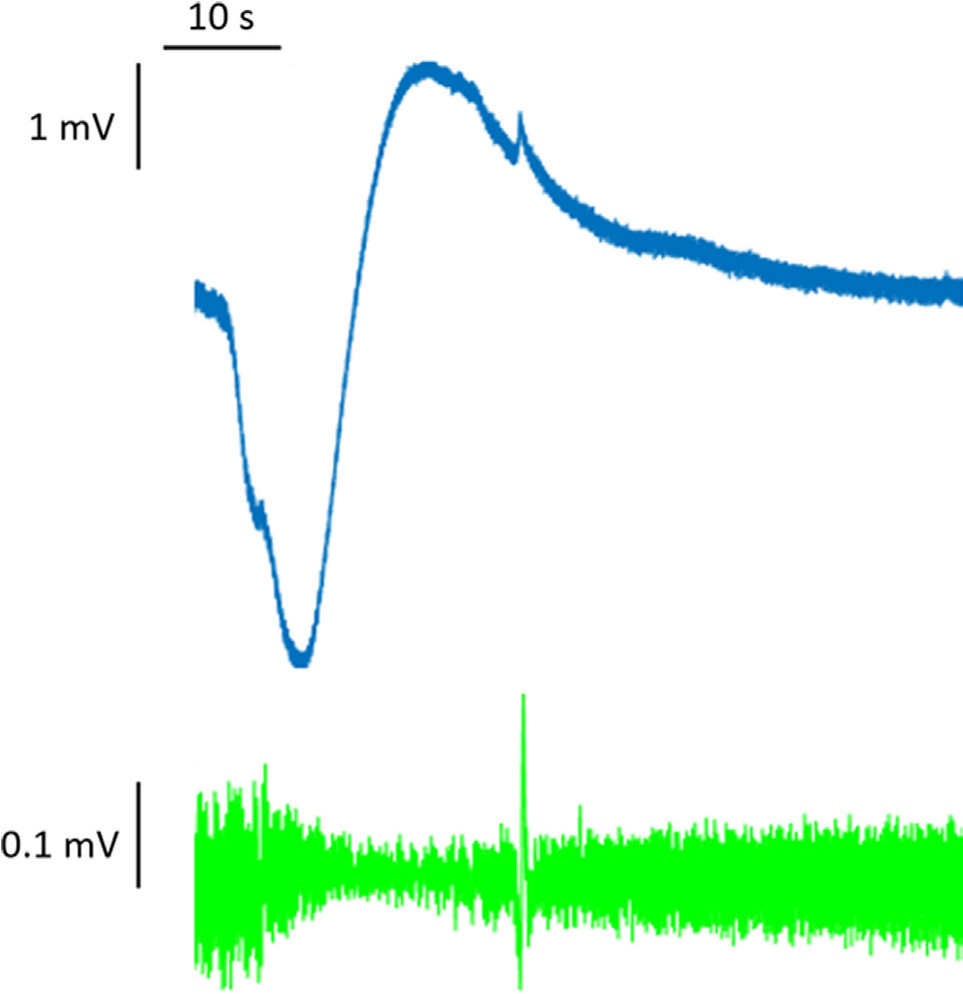
Full-band (DC-coupled) recordings of spreading depression. Two events propagate across the full electrode strip from electrodes 6 to 1, as shown by negative DC shifts and 0.5–50 Hz depressions of spontaneous activity. Analysis focused on electrode 3 in monopolar mode (electrode 3 versus reference, gold). Adapted from Ref. (14).

At the onset of an action potential, the membrane potential rises rapidly because of the opening of sodium channels, which temporarily generates an inward current that is not balanced by outward currents. By contrast, the slow temporal evolution of the depolarization typical of SD is due to a more gradual modification of the resting membrane potential, mediated by changes in intracellular and extracellular ion concentrations. Extracellular K^+^ concentrations, in particular, seem to be decisive in triggering and propagating the SD phenomenon. In physiological conditions, extracellular concentrations of K^+^ are relatively low, and the extracellular space is relatively small. K^+^ concentrations can increase rapidly by means of transmembrane fluxes (6, 7). Since the electroneutrality between the two sides of the cell membrane needs to be preserved and the membrane has a limited capacitance, changes in ion concentrations and sustained depolarization cannot result from a single ionic current but are mediated by sets of opposing currents. It is necessary to bear this concept in mind when seeking to interpret data from experimental studies aimed at blocking specific currents and investigating those involved in the SD phenomenon. SD is a process resting on breakdown of ion homeostasis that occurs when passive cation influx across cell membranes exceeds ATP-dependent Na^+^ and Ca^2+^ pump capacity (13). The massive discharge of the membrane potential seen in SD creates a signature shift of the extracellular potential that can be as great as −30 mV. This potential shift has a slow onset (many seconds), and it is difficult to detect with traditional high-pass AC amplifiers. To observe it, an unfiltered direct-coupled (DC) amplification is required; for this reason, it has classically been referred to as the DC shift (14) (Figure 1).

### Reduction of Local Electrical Activity

The propagating depolarization phenomenon results in a reduction of electrical activity that shows up as a marked negative slow potential change (4, 15, 16). This change may result from longitudinal gradients of depolarization along neurons (6). In fact, neurons do not seem to be inactivated along their entire anatomy during the massive cell depolarization; instead they maintain their integrity and electrical function, except in the dendritic zone where ion channel opening allows large sustained influxes of small cations, such as sodium and calcium (6). Intracellular potential gradients, from zero to rest, can be supported by a combination of shunted membranes and ion redistribution along discrete cell subregions.

The suppression of electrical activity in SD rests on reduction of synaptic currents that, in turn, reduces neuronal energy requirements. The cause of this reduction seems to be presynaptic. Synaptic failure is induced by high extracellular levels of adenosine, a breakdown product of ATP, which prevents the vesicular release of glutamate. Adenosine levels may rise as a result of increased ATP consumption (17, 18). Electrical activity remains suppressed for several minutes after repolarization, and neurons do not generate action potentials upon application of glutamate during this period (18). Recent experiments demonstrated that the large reduction in action potential firing observed after SD could be due to a shift in the excitation/inhibition ratio toward inhibition. They showed that reduced action potential firing, like post-synaptic potential amplitude changes, lasted at least an hour after the depolarizing event (19).

### Cell Swelling and Beading

Neurons have been found to show a morphological change during SD. Authors describe neuronal swelling and a transitory change in dendritic structures (“beading”) with an apparent loss of many dendritic spines (20). Extracellular volume decreases from 20 to ~5% because of the water influx due to ion changes, leading to intracellular hyperosmolality (21–24). The change in morphology of most dendrites is reversible and neuronal cell bodies were found to regain their previous volumes within 8–10 min after SD (20, 25–27). Moreover neurotransmitters, such as acetylcholine, γ-aminobutyric acid (GABA), and glutamate, are released in large amounts (28). Among these, GABA is particularly interesting as it can play two roles; indeed, GABA_A_ receptors contribute to limit the propagation rate (29), but may also facilitate cell swelling through chloride entry (30, 31). Indeed, blockade of Cl-coupled transporters leads to a significant reduction in dendritic beading without interfering with SD (32).

### Propagation

The depolarization propagates through the gray matter like a wave, typically spreading at a rate of 2–6 mm/min (15). Rather in the manner of an action potential, the depolarization wave, once triggered, propagates in an all-or-none fashion, regardless of the stimulus type or intensity. The exact propagation mechanism is not yet completely understood, even though some hypotheses have been advanced (33). Non-mutually exclusive mechanisms involved in the propagation may rely on the diffusion of extracellular potassium or glutamate, or alternatively on the opening of neuronal or glial gap junctions (12). However, the hypothesis of propagation based on regenerative glutamate release *via N*-methyl-d-aspartate receptor activation (28) runs into the problem that indirect calcium release from mitochondria seems insufficient to drive this process—depolarization in naive tissues has been found to be blocked by removal of extracellular calcium or inhibition of voltage-gated calcium channels (34–36)—while a transcellular pathway for diffusion *via* neuronal gap junctions (37) also seems highly unlikely, given that pyramidal cells display gap junctions only in early brain development (38), while in adult animals these junctions are present only between interneurons (39).

### Tissue Recovery and the Role of Astrocytes

Under normal oxygen conditions, electrical activity returns to its normal state within a few minutes after tissue depolarization, and then ion homeostasis is restored. Several mechanisms are involved in this tissue recovery: the Na/K-pump is activated as a result of increased intracellular sodium and extracellular potassium levels (40–42); repolarization of the neuronal membrane potential closes the voltage-gated channels, decreasing the potassium efflux and thereby allowing the pump to restore physiological ion concentrations. The repolarization is also promoted by the glial buffering of extracellular potassium in the extracellular space (43, 44). Under ischemic conditions, the compensatory action of astrocytes is hindered because astrocytic Na/K-ATPases lack ATP. Consequently, intra-astrocytic sodium rises (45), and potassium is spilled out instead of taken up (46). Loss of astrocytic function may severely limit neuronal survival under ischemia (47). SD is due, primarily, to disruption of neurons, while astrocytes remain functional and support neuronal recovery. In this process, changes in intracellular calcium, which rises first in neurons, then in astrocytes, suggest that neurons lead and astrocytes follow (48). Moreover, SD and the associated neuronal calcium wave remain unaffected when the astrocytic calcium wave is blocked by the depletion of internal calcium stores (35). However, SD involves a complex interplay between the activities of neurons, glia, and blood vessels. Interestingly, one study found that inhibition of astrocyte signaling did not abolish SD, although the changes in vascular caliber normally associated with SD were absent, suggesting that astrocytes play a key role in mediating the vascular response to the SD phenomenon, but not necessarily its propagation (48). Moreover, astrocytes swell markedly under ischemic conditions (26). Recent optogenetic studies have shown that SD can be elicited by specific stimulation of astrocytes. The finding that light stimulation of mice expressing a light-activated channel specific for astrocytes resulted in sustained depolarization of astrocytes and eventually elicited SD further adds to the evidence that astrocytes could have a primary role in SD initiation (49).

### Vascular Response

In healthy tissues, increased electrical activity is coupled with the release of vasodilator factors, such as nitric oxide (NO) and arachidonic acid metabolites, which increase local blood flow to meet increased energy expenditure (50). This system, known as “neurovascular coupling,” involves neurons, astrocytes, and arterioles (48, 51–53). In damaged tissues, on the other hand, the restorative vascular response is absent, and indeed there is a vasoconstrictor response (1, 13).

In the presence of extensive disruptions, such as SD, the vessel response is strongly nonlinear. While moderate increases in extracellular potassium cause vasodilation, stronger increases induce vasoconstriction (53). The neurovascular response to SD typically shows a triphasic pattern (constriction, dilation, and then prolonged slight constriction), but it differs greatly between species and conditions, ranging from pure constriction to pure dilation (54).

### Animal Models and Experimental Triggering

Experimentally, SD can be induced by various stimuli, including intense electrical stimulation, mechanical damage (needle prick), and administration of K^+^ or glutamate (55). Ultimately, these stimuli lead to glutamate-induced toxicity (21). Glutamate activates calcium and sodium channels, particularly the NMDA receptor channel, in a pathologic manner, causing the membrane potential to change from approximately −70 to −10 mV (13). Triggers of SD fall into two categories: those that depolarize neurons through sodium and/or calcium channel activation, and those that depolarize neurons indirectly *via* Na/K-ATPase activity reduction (7). Stimuli belonging to the first group include ictal epileptic events, administration of glutamate or potassium, and administration of neurotoxins, e.g., veratridine. The second group includes conditions associated with ATP depletion, such as ischemia, hypoxia and hypoglycemia, drugs such as ouabain or palytoxin, and brain topical superfusion of the vasoconstrictor endothelin-1 (56).

Spreading depression induced using transcranial stimulation of channelrhodopsin-2 ion channels was recently found to be similar to the SD changes evoked with KCl (57). Since different mechanisms of induction lead to different pharmacological effects, it is important to understand which experimental methods of SD induction are most relevant to specific human conditions (8).

### Genetic Influences

Gene mutations can decrease the threshold for the SD, by leading to spontaneous excessive localized increases in K^+^ or glutamate in some individuals. A genetic contribution to this phenomenon has emerged through study of, for example, mutations in the hemiplegic migraine-associated genes (58–62). Different genetically modified mice, characterized by astrocyte-directed inactivation of Cx43, showed a similar increased propensity to SD (63), since functional gap junctions are involved in spatial potassium buffering. Studies indicate that the propensity to SD in rodent models could also be related to gender (64), with female mice showing a lower threshold for induction of SD compared with their male counterparts (59, 65). The influence of sex hormones in this process is reported in different studies: SD susceptibility was found to increase in the presence of estrogen, whereas exposure to testosterone had the opposite effect (59, 66). This important sex-related effect might be due to the sexual dimorfism of chloride regulation as it has been shown that the main cotransporter responsible for chloride extrusion, KCC2, is downregulated by sex hormones in certain brain areas leading to less effective inhibition (67, 68).

## SD in Disease Conditions

A few studies have recently investigated the occurrence of SD as a characteristic of disease states in humans. The depolarizing phenomenon in SD is not substantially assessable by scalp EEG due to the filtering effect of the scalp, dura, and bone. Scalp EEG allows the detection of low amplitude and high frequency changes, like those seen during seizures, but not the slower, although higher amplitude, changes characteristic of SD (13). However, an attempt to find the scalp correlate of SD has been made through scalp EEG performed simultaneously with cortical surface recording using electrocorticography strips (69). Clinical monitoring of SD is currently limited to patients who need neurosurgical interventions necessitating the placement of subdural electrode strips (14, 69). SD events, recorded in humans, have electrophysiological features that are essentially the same as those observed in animal models. Furthermore, measurement of regional cerebral blood flow in migraine patients has revealed spreading oligemia whose scale and timing are similar to what is observed in SD (70).

### Migraine

Migraine is a common disorder characterized by recurrent attacks of severe, usually unilateral, headache accompanied by other symptoms (nausea and/or vomiting, photophobia and/or phonophobia). It is one of the most prevalent neurological disorders, affecting on average more than 15–20% of the population and showing a female:male ratio of 3:1. There are several subtypes of migraine; the two major ones are migraine with aura and migraine without aura (71). The migraine aura is a complex of neurological signs and symptoms that typically precedes headache onset by 20 min, and lasts 5–60 min before disappearing. More rarely, however, aura symptoms can accompany the headache for hours or even days. Auras consist mainly of transient visual, sensory, or language disturbances. Visual symptoms are the most common, and typically consist of unformed flashes of light moving across the visual field. Progression of the aura in migraine shows the typical spatial and temporal features of experimentally induced cortical SD in animals (56, 72). Moreover, waves of alterations in cortical activity and blood flow have been captured through functional imaging studies and found to show SD-like features both in migraineurs with and in those without aura (73–75). However, the link between SD and migraine without aura is still poorly understood, and it is not yet clear whether the SD runs over silent areas of the cortex, for example, subcortical regions (e.g., the hippocampus) (63), or whether, instead, it fails to reach the clinical threshold (76). Hemodynamic data are also conflicting, since some studies have shown no changes in cerebral blood flow during migraine attacks without aura, by contrast with the post-SD oligemia observed during auras in other research (77).

Cortical SD is now widely assumed to be the electrophysiological mechanism of migraine aura, and a large body of evidence supports this assumption. SD is the first endogenous event identified upstream to trigeminovascular activation leading to pain and blood flow changes. Although speculative, SD would emerge as a common pathophysiological mechanism causing pain and local autonomic changes, as well as hemodynamic patterns similar to migraine headaches in humans (78). A link between headache localization and the neurobiology of aura is provided by SD-induced activation and sensitization of neuropeptide-containing trigeminal peripheral nociceptors innervating ipsilateral cranial tissues (8). A drastic depolarization, as those occurring during cortical SD, will release noxious molecules such as different ions, i.e., H^+^ and K^+^, or NO, in the neocortical extracellular space (76). The rise in extracellular K^+^ during SD is thought to depolarize and excite the nociceptive fibers in the ophthalmic division of the trigeminal nerve enveloping the pial arteries, resulting in release, from the primary meningeal afferents, of pro-inflammatory peptides, i.e., calcitonin gene-related peptide and substance P (CGRP), that induce vasodilation and plasma protein extravasation, thus triggering the headache (77, 79, 80).

This theory has been confirmed by the increased pro-inflammatory peptide levels seen in patients with migraine attacks (81) as well as in electrically stimulated rats (82). Repetitive episodes of SD, moreover, have been found to induce inflammatory cascades that resulted in the production of cyclooxygenase 2 and inducible NO synthase, and in the activation of microglia (82, 83). The clearance of extracellular macromolecules is thought to be mediated mainly by the glymphatic pathway, a system consisting of astroglial cells forming series of perivascular compartments that clear waste products from the brain (84). Significantly (from the perspective of the migraine pathomechanism), it has recently been demonstrated that SD impairs glymphatic flow, thus interfering with the clearance of excitatory and inflammatory chemicals and might foster localized cortical hyperexcitability and structural thickening (85). SD, therefore, by supporting inflammatory responses and the consequent activation and sensitization of meningeal trigeminal afferents, seems to play a strong role in headache pathophysiology. A mutually causative link between inflammation and SD is suggested by the recent observation that maternal inflammation interferes with chloride regulation of the off-springs, delaying the switching from depolarizing (high intracellular Cl) to hyperpolarizing GABA (86) (low intracellular Cl). If the connection between inflammation and chloride control was established also in adults, SD and inflammation might constitute a self-supporting system that may lead to a gradual worsening of the condition.

The exact mechanisms of brain dysfunction leading to the onset of a migraine attack, as well as the determinants affecting individual susceptibility to SD in the human brain, remain unknown. Genetic factors predisposing to migraine with aura also enhance susceptibility to SD (87). Most studies exploring these aspects involve transgenic mice expressing mutations associated with the different forms of familial hemiplegic migraine (FHM), or with cerebral autosomal dominant arteriopathy with subcortical infarcts and leukoencephalopathy (88, 89). Migraine is multifactorial and its rare monogenic forms (i.e., FHMs) phenocopy most or all the clinical features of the classic form of the disease, although they typically show a longer duration of auras as well as the presence of motor features (90). Three main FHM genes have been identified: *CACNA1A* (FHM1) encoding the alpha subunit of the neuronal voltage-gated Ca^2+^ channel (Cav2.1) (91), the *ATP1A2* gene (FHM2) encoding the alpha-2 subunit of a Na^+^/K^+^ pump (61), and the *SCN1A* gene (FHM3) whose mutations result in defects of the alpha-1 pore-forming subunit of the neuronal voltage-gated Na^+^ channel (Nav1.1) (92).

Acetazolamide, used to treat headaches associated with FHM, has been shown to decrease the frequency of attacks, and its effectiveness has been suggested to be due to improvements in ion channel function (93, 94). In the central nervous system, acetazolamide penetrates the blood–brain barrier slowly and causes carbonic acidosis through carbonic anhydrase inhibition (95); this acidosis may also serve to selectively reduce the buffering of rapid pH changes occurring in SD. Indeed, at the onset of SD, there is a brief extracellular alkaline shift (96) that transiently promotes neuronal injury by eliminating the proton block of NMDA receptors (97). Transgenic mice expressing either FHM1 or FHM2 mutations show both enhanced susceptibility to SD and altered synaptic transmission, thus highlighting possible pathophysiological mechanisms underlying FHM (98). However, whether these mutations also predispose to typical migraine remains poorly understood (99). Finally, there is evidence to suggest that sex hormones may modulate SD susceptibility in humans (100), as well as in FHM animal models (66). Sex hormones may enhance susceptibility to SD in FHM1 female mice, and this increased susceptibility may recover after oophorectomy or estradiol treatment (59, 66). These findings may, in part, explain the increased prevalence of migraine in females and its variability across the individual life span.

Different classes of prophylactic drugs for migraine, such as antiepileptic drugs (topiramate, valproate, and lamotrigine), as well as propranolol, amitriptyline and methysergide, are able to suppress SD, through different mechanisms of action (101), and they are equally efficacious both in forms with and without aura (102). Lamotrigine, in particular, targeting Ca^2+^ and Na^2+^ channels and glutamatergic or GABAergic transmission (mechanisms common to most antiepileptic drugs), has been shown to be effective in the prevention of migraine aura possibly through an inhibitory effect on SD and a consequent suppressive effect on the aura phenomenon (103, 104). Furthermore tonabersat, a relatively novel benzopyran derivative known to inhibit gap-junction communication between neurons and satellite glial cells in the trigeminal ganglion (105) and to markedly reduce SD-associated events in preclinical studies (106) has been proposed in migraine prophylaxis and to relieve headache in migraineurs (107). However, results of randomized, double-blind, placebo-controlled studies showed benefit on attacks of migraine aura but no effects in reducing migraine pain days when chronically administered to patients (108). Results in acute studies are conflicting.

Administration of the serotonin precursor 5-hydroxytryptophan in animal models has also been shown to decrease the frequency of KCl-induced SD in female rats, suggesting a possible role for serotonergic agents as therapeutic tools in migraine with aura in females (109). Blocking the SD mechanism, therefore, seems to be a rational approach to explore in molecular research aimed at improving the prevention and treatment of migraine with aura. However, the evidence that different symptoms of migraine may already be present at the time the visual aura occurs, and the fact that aura can be painless in some migraineurs, confirm that the real role of SD in migraine is still to be entirely clarified.

New approaches to migraine treatment were recently developed adopting fremanezumab, a humanized monoclonal antibody (CGRP-mAb) able to reduce the availability of CGRP. Fremanezumab pre-treatments prevents headache by selectively inhibiting the responsiveness of Aδ neurons (peripherally) and high threshold neurons (centrally), but not C-fiber neurons, as reflected in a decreased percentage of neurons showing activation by SD. The selective inhibition of fremanezumab on meningeal nociceptors might explain why a CGRP-mAb may not be effective in all migraineurs (110). Another CGRP receptor antagonist, MK-8825, dose dependently seems to attenuate SD-induced trigeminal nerve mediated pain response but without altering SD waves (111).

#### Other Migraine Disorders

It is worth noting that the International Classification of Headache Disorders, third version (71) lists features for other less frequent migraine disorders, such as status migrainosus, persistent aura without infarction, and migrainous infarction (71). The aforementioned SD mechanisms may play a significant role in these conditions, as demonstrated specifically in migrainous infarction, a condition that may lie on a continuum with cerebral ischemia (see below) (112–114). It has been shown that FHM1 mutations may share genetic determinants of migraine with aura and stroke; indeed, these mutations, typically associated with migraine with aura, increase stroke vulnerability and also accelerate stroke evolution (115) by enhancing the susceptibility to ischemic depolarizations, a process akin to SD. In addition, in a subset of migraineurs, the presence of small microemboli could trigger SD and thus possibly be a stimulating mechanism for aura even in the absence of tissue injury (116).

### Ischemia and Subarachnoid Hemorrhage

The term ischemia (or stroke) refers to a decrease in blood supply to a tissue or organ. In focal cerebral ischemia, in particular, cerebral blood flow reduction is confined to a single anatomic area of the brain (117, 118), and has a number of effects on the surrounding nervous tissue, including excitotoxicity, oxidative stress, and apoptosis (119). In the early phase of ischemia, tissue damage is primarily related to direct metabolic failure caused by the decreased blood flow (120); this leads to impairment of ionic pumps (7), which in turn results in ionic imbalance and accumulation of cytosolic sodium and calcium ions and extracellular potassium (121), causing water influx into cells, tissue swelling, and permanent depolarization of cell membranes. Immediately after the acute phase, the damage spreads, over several hours, to the adjacent areas, or ischemic penumbra (122), i.e., to a wider area of tissue that also becomes severely compromised by the blood flow reduction (123). This process is driven by a plethora of electrochemical events, so-called peri-infarct depolarizations (PIDs) that produce multiple waves of cortical SD-like activity and are fundamental mechanisms in the continuous elicitation of the biochemical cascade of cell injury. While SD is not directly linked to cell death or damage in a normally perfused brain, recurrent PIDs in an ischemic brain are associated with enlargement of the infarcted volume (13, 124, 125).

The features PIDs are similar to those of SD, with extracellular DC shifts of 20 mV, a propagation velocity of 3–5 mm/min, and disruption of the cellular ion balance. In all animal studies of focal ischemia, the vascular responses of ischemic tissue and adjacent areas were seen to vary considerably, depending on distance from the ischemic focus (126–128). In more severely ischemic areas PIDs mainly cause monophasic hypoperfusion; instead, where ischemia is milder, the response becomes biphasic, with an initial hypoperfusion followed by peak hyperemia. In non-ischemic adjacent areas, PIDs cause only hyperemia, and there is no hypoperfusion (129, 130). NO may play a role (129, 131), since the reduction in O_2_ availability, due to the ischemic condition, may in fact reduce NO synthase. This would result in a diminished NO-dependent vasodilation. Another factor is the extracellular concentration of K^+^ that, in the injured brain, is usually higher than normal. High concentrations of K^+^ are linked to a stronger vasoconstriction (132).

Spreading depression has also been observed in the brains of patients affected by cerebral ischemia, and in these subject PIDs, similarly to those measured in animals and associated with different hemodynamic responses, such as hyperemia, biphasic response, or hypoperfusion, have been linked to increase of the infarcted volume (133, 134).

Taken together, animal models and human studies show that SD-like depolarizations, i.e., PIDs, may represent a core mechanism for stroke pathophysiology, clearly related to the expansion of the infarcted volume and the spread of ischemia (83).

About 10–20% of all cases of stroke, however, result from subarachnoid hemorrhage, usually originating from the rupture of an aneurysm (135). After the occurrence of a subarachnoid hemorrhage, delayed ischemia affects the injured brain and can cause fatal damage or permanent injury (136). SD may play a pivotal role in this delayed cerebral ischemia and contribute to its diffusion (137). Although SD has been observed in animal models of brain hemorrhage, its precise role has never been demonstrated in a clear and reproducible manner, and therefore it remains debated (138, 139). For example, local superfusion of hemoglobin in rat brains, together with elevated concentrations of extracellular K^+^, induces SD with a monophasic vasoconstriction response. Hemoglobin is indeed thought to reduce NO concentration, by acting as a scavenger (140). Moreover, the superfusion of hemoglobin or NO synthase inhibitors, with the addition of either endothelin-1 or a small amount of glucose, is sufficient to produce SD and the spreading of ischemia (141).

Spreading depression after subarachnoid hemorrhage has also been observed in humans, accompanied by an increase in the infarcted tissue volume and delayed ischemia (16). In subarachnoid hemorrhage, as in ischemia, SD and PIDs may be worsening factors, given their detrimental impact on already damaged brain tissue. They have a dual mechanism of action: increasing metabolic activity and reducing blood supply.

### Traumatic Brain Injury

In recent clinical studies, SD has emerged as a potent pathomechanism of the progression of secondary injury in TBI (142–144). The incidence of SD among TBI patients is reported to be around 50–70%, and SD often occurs in repetitive patterns over a period of at least 7 days post-trauma (143, 144). Although the probability of SD is theoretically increased as a result of lower levels of cerebral perfusion and high systemic temperature, which lead to an increased mismatch between energy supply and demand (145), the vast majority of SD in TBI occurs when the systemic variables are in the normal range (142). In post-TBI patients, who underwent craniectomy, the EEG was able to track 40% of depolarizations, recorded simultaneously with cortical electrodes (144). Following TBI, increased glutamate levels trigger SD (146–148). In addition, astrocyte activity increases after TBI (149) while the expression of GLT-1, a glutamate transporter, is reduced (146). It remains unknown how an acute cortical lesion might, in relation to the site of the injury, affect the site of initiation and subsequent propagation of SD. The cortical injury site is not capable of initiating SD, and the depolarization takes place in the surrounding peri-injury zone. Instead, the injury zone has been found to act as an attractor, having the ability to support SD initiated in nearby, less compromised networks (150).

### Epilepsy and Sudden Unexpected Death in Epilepsy (SUDEP)

In addition to its possible roles in migraine and cerebral ischemia, subarachnoid hemorrhage, and TBI, SD has been also related to epileptiform activity (13, 56, 151–153). SD, as we have seen, is a disruption of electrical activity caused by a strong ion concentration change in the extracellular space. The impaired ion homeostasis associated with SD triggers a cascade of cellular events leading to toxic release of glutamate (21). From this perspective, excitotoxic states emerge as a mechanism common to both SD and seizures, in the former caused by neuronal activity depression, and in the latter by neuronal hyperactivation.

Because of this shared background, there exists a complex and as yet poorly understood interrelation between epileptic activity and SD. Several studies have shown that seizures can either potentiate or limit SD waves, depending on whether they occur before, during or after the SD event. Some authors, examining brain slices from individuals with epilepsy, showed that their tissue was highly susceptible to generating SD waves (154). Similarly, studies of animal models of seizures, induced pharmacologically, have shown an increase in SD wave susceptibility after seizure events (155, 156). The depression of neuronal electrical activity caused by depolarizing waves may be the cause of the post-ictal depression state typically seen in epilepsy. Multiple SD waves have been shown to occur during post-ictal periods, providing a possible explanation for the observed slow post-seizure cognitive recovery (155). Looking at the question from the opposite perspective, SD may create an environment conducive to the onset of epileptiform activity. Both mouse and human experiments have shown states of hyperexcitability following a cortical depression period, akin to that seen prior to epileptic seizures (157–159). An explanation for this may be related to the neuronal swelling that occurs following SD waves, which may increase neuronal excitability through activation of NMDA receptor-dependent inward currents (160). Furthermore, a change in the pattern of neurotransmitter receptor distribution has been noticed after cortical SD events, and this may in turn imply a lowering of the seizure threshold (161). Contrary to this potentiation effect, SD is also capable of interrupting a seizure, even leading to refractory periods of seizure silencing in rats (155, 162, 163). Conversely, stimulation-induced recurrent epileptiform discharges have been shown to block cortical and subcortical SD in rats (164). These findings do not allow a single interpretation of the interplay between SD and epilepsy, but they strongly suggest that there is a common mechanism in which a pivotal role may be played by NMDA receptors (165).

It has also been suggested that the process of SD could underlie failure of the brain to recover following a seizure (166). Seizures are typically accompanied by transient alterations in cardiac and respiratory rates, but these events are generally reversible as soon as the seizure ends. However, SUDEP, a condition where death is caused by seizure-induced cardiorespiratory collapse, is among the leading causes of mortality associated with epilepsy-related disorders. Although it accounts for about 17% of epilepsy-related deaths (167) (~50% among cases of refractory epilepsy) (168, 169), there is still no consistent and strong body of literature on the topic, containing comparable data linking SUDEP with its risk factors. This knowledge gap is probably attributable largely to the great heterogeneity of research approaches, the multifactorial nature of SUDEP, and also the impracticality of following a large number of individuals with epilepsy from diagnosis to death. Furthermore, investigation of SUDEP is also complicated by the fact that the exact mechanism underlying this phenomenon remains to be fully elucidated.

Studies both in humans and in mice (170–172) have suggested that a genetic misregulation of brain centers controlling autonomic functions, resulting in alterations in membrane excitability or synaptic activity in pathways that control heart rate or agonal respiration, is the basis of increased risk of SUDEP (173, 174). Moreover, several mutations in genes linked to increased SUDEP susceptibility have been demonstrated to be directly linked to a lower threshold for SD in mice (170, 171). Against this background, Aiba and Noebels (166) have proposed that the SD phenomenon is the causative process leading to cardiac and respiratory shutdown in susceptible individuals (175). If triggered in the brainstem, the depolarizing electrical wave may diffuse across cardiorespiratory centers in the medulla, leading to complete arrest of pace-making neuronal centers, hindering recovery of heartbeat and normal respiration after a seizure and leading to death (166). In the same study, cortical seizures, obtained by cortical application of 4-aminopyridine, were found to be capable of causing a slowly spreading negative DC potential which also can diffuse down to the dorsal medulla.

Two relatively recent genetic mouse models, Kv1.1 potassium channel and Scn1a sodium channel KO animals, which replicate many aspects of human SUDEP, have provided strong indications that SD acts as an intermediary between a severe seizure event and consequent cardiorespiratory failure leading to sudden death. Functional alterations of these genes impair neuronal excitability through different membrane and synaptic mechanisms (166, 174, 176). Ablation of the Kv1.1 gene has a strong effect on both excitatory and inhibitory neurons, whereas lack of the Scn1a channel reduces forebrain interneuron excitability (177–180). In both cases, the result is disruption of the excitatory/inhibitory equilibrium and a dramatic lowering of the threshold for triggering SD waves. As a consequence, whereas these animals recover normally if seizures remain below this threshold, severe seizures can trigger an SD event, leading to death within minutes (166, 176). This mechanistic interpretation of SUDEP may explain why epileptic individuals with seizure-induced cardiorespiratory arrest may fail to be revived using conventional cardiopulmonary resuscitation procedures, and suggests a possible therapeutic target for this fatal complication of epilepsy.

## The “Dark Side” of SD

In the past few years, the main hindrance to the study of SD was the difficulty in detecting depolarization wave non-invasively in the human brain (181). On the other side evidence associates SD to a plethora of phenomena, from relatively benign migraine aura attacks to severe brain injuries, such as ischemic strokes, subarachnoid or intracerebral hemorrhage, and SUDEP. This heterogeneous presentation increases the interest on SD as a pathological mechanism of great importance for clinical neuroscience. The “dark side” of this process is that it is hard to explain the heterogeneity of SD and to predict *ab initio* its severity. As already speculated by others, the mechanisms underlying the different clinical outcomes of SD can depend on several factors. One of these is the specific brain “status” based on the patient’s vascular and metabolic conditions, an as yet vague genetic predisposition, and eventually preexistent pathological conditions (13, 50). For instance SD is a relatively benign phenomenon in migraineurs because it occurs in normal human brains with preserved perfusion and energy metabolism. Conversely, in injured brain with compromised energy metabolism and perfusion, one could hypothesize that SD might contribute to further brain damage, such as expansions of brain infarcts, and could impair clinical recovery, or trigger new deficits. Yet, other factors need to be determined in the frequency, properties, and impact of SD, including the role of chronic medications and variations in plasma osmolality, and serum levels of electrolytes and glucose (147). In sight of these speculations, we need further understanding on how perturbed tissue will affect the expression and the consequences of SD.

## Conclusion

**Figure 2:**
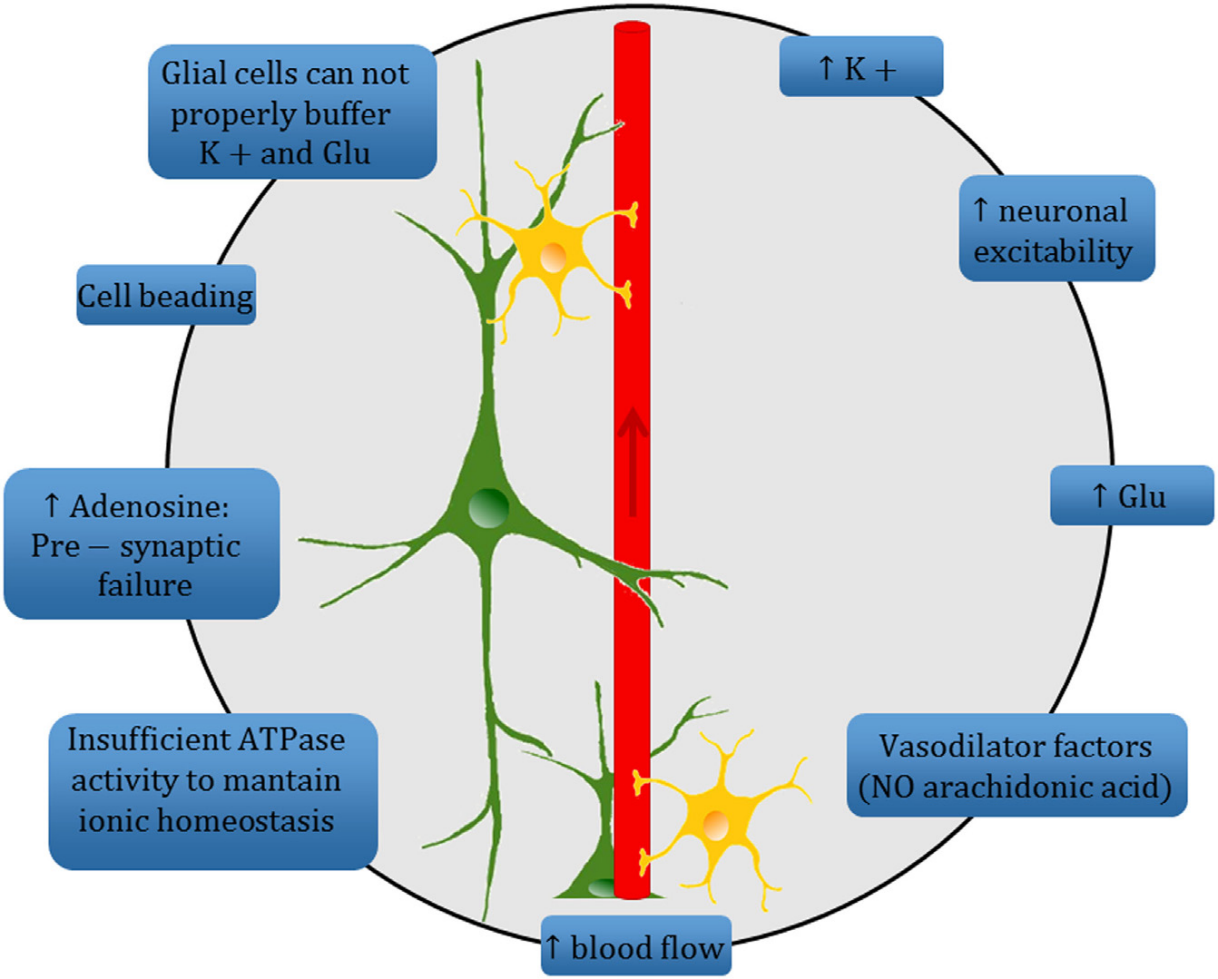
Schematic diagram summarizing the complex mechanisms underlying the spreading depression (SD) phenomenon at the tripartite synapse. High extracellular K^+^ concentration causes an increase of neuronal excitability and a high level of extracellular Glu. Increased electrical activity leads to release of vasodilator factors with a consequent increase in blood flow. ATPase activity is insufficient to maintain ion homeostasis and leads to high level of adenosine which, in turn, prevents vesicular release of Glu at presynaptic level. A change in dendritic structures and the loss of dendritic spines occur («beading»). The astrocyte-mediated buffering of K^+^ and Glu is inadequate to quickly restore the electrochemical homeostasis, and thus sustains the SD mechanism.

The ambiguous interplay between SD and the various related clinical phenotypes precludes univocal identification of its precise role in brain disease. Even though it is often considered a single phenomenon with homogeneous characteristics—its signature features being slowly propagating changes in neuronal electrical potentials and silencing of brain electrical activity, together with neuronal swelling and distortion of dendritic spines (147)—SD shows some heterogeneity in its cellular and molecular mechanisms (8), and appears to play different roles in different clinical disorders (as an event-triggering process, secondary effect, or simple epiphenomenon) (Figure 2). Further advances in our knowledge of this phenomenon are necessary in order to improve our understanding of these different conditions, since better disentangling of the underlying processes may also foster the development of targeted interventions able to prevent their most dramatic consequences.

However, as well as studying the specific mechanisms characterizing the different disorders, it is still necessary and may well be useful to focus, also, on the common features of SD in the various conditions. A number of models of genetically determined migraines (like FHMs) or epilepsy, such as Kv1.1 potassium channel and *Scn1a*^−/−^ animals (177–180), are already widely available and may serve as useful, paradigmatic tools able to disclose the core mechanisms of the SD phenomenon, common to the different clinical disorders. This might help to identify new treatment targets, possibly making it possible to overcome the traditional, only partially efficacious, therapeutic approaches, i.e., with classic antimigraine or antiepileptic drugs, and open new perspectives for treating these disorders or preventing their worst consequences.

Despite the development of several animal models for the study of SD, many questions remain open as a result of its complexity and the difficulty of obtaining direct experimental data in humans. In addition to more detailed characterization of existing or new models of spontaneous SD, which may be useful for dissecting and clarifying the pathomechanisms in animals, we need to continue developing non-invasive technologies (182) that might allow us to gather additional information in humans, since, for the moment, subdural strips continue to be the main means of monitoring SD in this setting.

## Author Contributions

All the authors have contributed substantially to the writing and revising of the manuscript. OC, MM, FT, FS, and FMS participated in the conception and design of the work, collected the literature, prepared the figures, and wrote the manuscript. EP, GR, and MB reviewed and edited the manuscript, and approved the final version.

## Conflict of Interest Statement

The authors declare that the research was conducted in the absence of any commercial or financial relationships that could be construed as a potential conflict of interest.
